# On the spatiotemporal dynamics and couplings across epileptogenic networks

**DOI:** 10.1186/1471-2202-14-S1-P196

**Published:** 2013-07-08

**Authors:** Timothée Proix, Viktor Jirsa

**Affiliations:** 1 Institut de neuroscience des systèmes, Inserm UMR 1106, 13005 Marseille, France

## 

For patients with partial epilepsy, one or several brain regions generate seizures ('epileptogenic zone' [1]), which may recruit other regions that are themselves non-epileptogenic. For these patients, the epileptogenic zone can sometimes be surgically removed and thus must be precisely delineated using intracranial EEG, giving us a deeper insight into the spatiotemporal dynamic of the seizure.

Intracranial EEG times series show that brain regions and seizures may display a large variability in dynamics: (i) some seizures recruit more brain regions than others, (ii) the delays between the onset of the seizure in the epileptogenic zone and other areas can change on a time scale of several seconds, (iii) recruited areas exhibit different levels of coherence with the epileptogenic zone according to their position.

**Figure 1:**
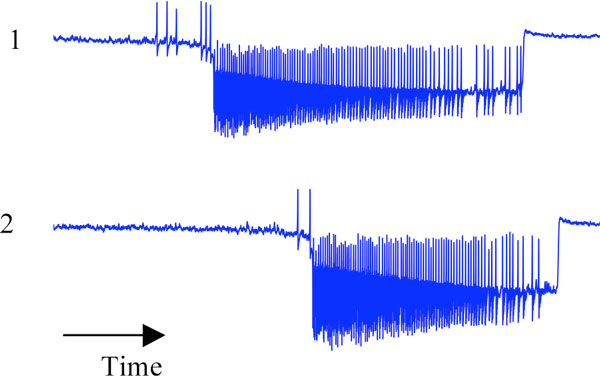
**Simultaneous times series of two coupled neural masses**. The neural mass 1 starts first the seizure and then recruits the neural mass 2.

Here we propose a model able to reproduce these 3 features (for other macroscopic models of seizures see [2,3]). Our network model comprises two neural masses autonomously able to produce epileptic seizure. We symmetrically coupled two of these neural mass models on a slow time scale (several seconds) and studied systematically the effect of two parameters : the epileptogenicity of the neural masses and the coupling values between neural masses. By choosing specific values of these parameters, we reproduce each of the features above (see Figure 1). We mathematically reduced the network model to uncover the essential mechanisms underlying a coupling acting on a slow time scale.
